# Estimating the Responsiveness and Minimal Important Change of the Trunk Impairment Scale in Inpatients With Subacute Stroke Requiring Dependent Ambulation

**DOI:** 10.7759/cureus.82275

**Published:** 2025-04-14

**Authors:** Naoya Yamamoto, Tatsuya Igarashi, Rin Sato, Hikaru Yamaoka, Sho Maruyama

**Affiliations:** 1 Department of Rehabilitation, Shonan Keiiku Hospital, Fujisawa, JPN; 2 Department of Physical Therapy, Faculty of Health Science Technology, Bunkyo Gakuin University, Fujimino, JPN; 3 Graduate School of Medical Sciences, Kitasato University, Sagamihara, JPN; 4 Department of Occupational Therapy, Graduate School of Human Health Sciences, Tokyo Metropolitan University, Hachioji, JPN; 5 Occupational Therapy Course, Department of Rehabilitation, Kitasato University, Sagamihara, JPN

**Keywords:** minimal important change, rehabilitation, responsiveness, stroke, trunk

## Abstract

Introduction

The Trunk Impairment Scale (TIS) has gained attention as a comprehensive tool for evaluating static and dynamic sitting balance, as well as trunk coordination. In particular, the responsiveness and minimal important change (MIC) of the TIS, specifically in patients with subacute stroke requiring dependent ambulation, have not yet been clearly established. The primary objective of this study was to determine the responsiveness and MIC of the TIS in patients with subacute stroke requiring dependent ambulation. A secondary objective was to compare the measurement properties of the TIS with those of the Trunk Control Test (TCT), a simpler and widely used tool, in order to identify which is more appropriate for detecting changes in trunk function in this specific patient population.

Methods

A retrospective cohort study was conducted involving 32 patients with subacute stroke. Eligible participants were diagnosed with cerebral infarction, cerebral hemorrhage, or subarachnoid hemorrhage and had a Functional Ambulation Category (FAC) score of 3 or lower, indicating a need for assistance with ambulation. All participants were assessed at admission and approximately one month later using the TIS, TCT, and Berg Balance Scale (BBS). MIC was calculated using receiver operating characteristic (ROC) curve analysis. Patients who improved by 5 or more points on the BBS were classified as the "important change group", while those with less than a 5-point improvement were classified as the "non-important change group". Responsiveness was analyzed through Spearman's correlation for score changes, skewness analysis for ceiling and floor effects, and unpaired t-tests between groups. MIC was calculated only when both the correlation coefficient (ρ) was ≥0.3 and the area under the ROC curve (AUC) was ≥0.7.

Results

The change in TIS scores differed significantly between the important and non-important change groups (p=0.001), and a moderate correlation was observed with BBS score changes (ρ=0.495; p<0.001). Skewness analysis showed no ceiling effect for the TIS. The AUC of the ROC curve for the TIS was 0.776 (95% CI: 0.612-0.941), and the MIC was calculated to be 2.5 points (95% CI: 0.5-4.0). In contrast, the TCT showed no significant difference in change scores (p=0.968). Ceiling effects were observed at both admission (37.5%) and follow-up (43.8%), the correlation with BBS was low (ρ=0.197; p=0.280), and the AUC was also low at 0.518, failing to meet the criteria for MIC calculation.

Conclusion

This is the first study to evaluate the responsiveness and MIC of the TIS in a clearly defined population of patients with subacute stroke who require assistance with ambulation. The MIC of 2.5 points observed in this study may serve as a potentially useful reference for clinical interpretation. Moreover, the findings suggest that the TIS is a more useful assessment tool than the TCT for evaluating trunk function in patients with subacute stroke requiring ambulation assistance.

## Introduction

Stroke results in various sequelae, including motor paralysis, sensory disturbances, and cognitive dysfunction. Among these, trunk dysfunction frequently leads to loss of independence [1]. Declines in trunk function are associated with impairments in balance, gait, and upper limb function [2]. Additionally, sitting balance, a key component of trunk function, is recognized as a strong predictor of motor recovery after stroke [3].

The Trunk Control Test (TCT) [4] is widely used to assess trunk function. The TCT evaluates four basic movements in bed (turning to the affected/unaffected side, sitting up, and maintaining a sitting position) and has demonstrated reliability and validity. Collin and Wade [4] reported a correlation between TCT scores and ambulation in patients with stroke. However, the TCT does not account for compensatory strategies, and its low difficulty level may lead to a ceiling effect [5].

In recent years, the Trunk Impairment Scale (TIS) has gained attention as a potentially more comprehensive tool for trunk function assessment. Developed by Verheyden et al. in 2004, the TIS evaluates static and dynamic sitting balance, trunk coordination, and movement quality, providing scores from 0 to 23 [6]. The TIS has demonstrated high reliability and validity and is widely adopted internationally as a detailed measure of trunk function. The Academy of Neurologic Physical Therapy recommends the TIS in clinical practice guidelines for neurological conditions [7].

The effective clinical implementation of tools such as the TCT and TIS requires a thorough understanding of their psychometric properties. Key attributes include validity, reliability, responsiveness, and interpretability [8]. Among these, responsiveness reflects the tool's ability to detect changes over time and accurately capture improvements in patient function. Interpretability refers to the extent to which numerical scores can be translated into meaningful clinical insights. One important aspect of interpretability is the minimal important change (MIC), the smallest difference in score that patients perceive as meaningful or beneficial. MIC has gained increasing relevance in rehabilitation, serving as a critical benchmark in clinical evaluations [9].

The MIC and responsiveness of the TIS in patients with stroke have previously been reported by Monticone et al. [10]. However, Revicki et al. have emphasized that MIC values should not be determined based solely on a single study but should be validated across multiple studies, even within the same disease or stage [11]. For instance, the MIC calculated for an asthma-specific quality-of-life measure in patients with mild to moderate asthma may not be generalizable to clinical trials involving patients with more severe asthma. This highlights the importance of considering patient characteristics when determining MIC values, even within the same condition or stage [11,12]. In the study by Monticone et al. [10], the MIC was calculated using the anchor-based method, while the distribution-based method was not employed. Moreover, the inclusion criterion for participants was the ability to maintain a sitting position, suggesting that the study population may have been broad. Therefore, caution should be exercised when generalizing these findings.

Furthermore, a recent study has suggested that the MIC may vary depending on the severity of ambulatory function. Smith and Patritti demonstrated that MIC values differed between patients with Functional Ambulation Category (FAC) level 3 and those with higher FAC levels, indicating that gait severity should be taken into account when interpreting MIC [13]. Therefore, limiting the study population and providing additional data may contribute to enhancing the robustness of MIC estimates.

We hypothesize that the TIS shows better responsiveness and interpretability compared to the TCT in patients with subacute stroke requiring dependent ambulation. If supported, this hypothesis could contribute to improving the assessment of trunk function and inform clinical decision-making in stroke rehabilitation settings.

The purpose of this study was to estimate the responsiveness and MIC of the TIS in inpatients with subacute stroke who require assistance for ambulation, with the goal of improving the robustness of MIC values. Additionally, we aimed to compare the TIS with the TCT, a commonly used measure of trunk function, to evaluate whether the TIS is appropriate for assessing intervention effects.

## Materials and methods

Study design and samples

This study was conducted as a single-center retrospective cohort study, aiming to examine a pre-specified hypothesis established prior to data analysis. Patients with stroke admitted to the convalescent rehabilitation ward at Shonan Keiiku Hospital between March 2023 and August 2024 were included. The study population was limited to consecutive cases that met clearly defined inclusion criteria. Furthermore, subject selection was rigorously conducted in accordance with a pre-established study protocol. Inclusion criteria were as follows: (1) patients admitted with a first-ever occurrence of stroke (cerebral infarction, cerebral hemorrhage, subarachnoid hemorrhage), (2) patients with dependent ambulation (FAC ≤3) [14], and (3) patients with sufficient cognitive function to adequately understand and appropriately perform the instructions for all test procedures. Exclusion criteria were the following: (1) patients with significant medical conditions or severe comorbidities, (2) patients with missing data, and (3) patients who withdrew from the study during the opt-out period. All patients participated in a rehabilitation program that included range of motion exercises, muscle strengthening exercises, balance exercises, gait training, and activities of daily living (ADL) training for at least 60 minutes per day. Appropriate sample sizes for receiver operating characteristic (ROC) curves were calculated using R for Mac Version 4.4.1 (R Foundation for Statistical Computing, Vienna, Austria). For each ROC curve result, assuming an area under the curve (AUC) of 0.8, a power of 0.9, and a proportion of both groups of 50%, the minimum number of cases required per group was determined to be 14.

This study was approved by the Ethics Committee of Shonan Keiiku Hospital (approval number: 24-016). Patient data were collected from medical records and databases, and all patients were provided with the opportunity to opt out if they wished to withdraw their data. This study was conducted in accordance with the Strengthening the Reporting of Observational Studies in Epidemiology (STROBE) guidelines [15] and the COnsensus-based Standards for the selection of health status Measurement INstruments (COSMIN) [16].

Data collection procedures

Patient characteristics, including age, height, weight, gender, stroke type, time since stroke onset, affected side, Mini-Mental State Examination (MMSE), and FAC, were collected from medical records. TIS, TCT, and Berg Balance Scale (BBS) scores were recorded at baseline (within two weeks of admission) and at follow-up (approximately one month after the baseline assessment). A previous study has suggested that recovery of trunk function in patients with stroke tends to be more pronounced in the early phase after onset [17]. Therefore, in this study, we set the follow-up period to approximately one month from the baseline assessment conducted at admission.

The FAC, TIS, TCT, and BBS were assessed by experienced physical therapists involved in daily clinical practice. The data used in this study were obtained from standardized quantitative assessments that were routinely recorded in electronic medical records as part of daily clinical practice. During the study period, no substantial changes were made to the evaluation system, evaluator composition, or assessment criteria.

FAC uses a 6-point scale (0-5), with higher scores indicating greater ambulation independence [14]. Patients with dependent ambulation (FAC ≤3) requiring supervision or assistance were included.

The TIS is a tool developed to assess trunk function in patients with hemiplegia after a stroke. It consists of three sections (static and dynamic sitting balance, trunk coordination), and the total score ranges from 0 to 23 points [6]. Each sub-item is as follows: For static sitting balance, maintain a resting sitting position, the therapist and patient each cross the unaffected leg over the hemiplegic leg to maintain a sitting position, and evaluate the degree of independence of each movement. For dynamic sitting balance, the patient touches the elbow of the hemiplegic side and the non-hemiplegic side to the bed or table located on the side of the body, respectively, and returns to the starting position. Each movement is assessed for its degree of independence, reproducibility, and the occurrence of compensatory movements. Lift the pelvis off the bed or table on the hemiplegic and non-hemiplegic side and return it to the starting position. Evaluate the independence and reproducibility of this movement. For trunk coordination, evaluate the symmetry and smooth reproducibility of six repeated rotations of the upper and lower body in a seated position [6]. Each item is scored on a scale of 0-2 or 0-3, with higher scores indicating better trunk function. The TIS has been reported to have good reliability and validity for measurement in patients with stroke [6].

The TCT measures trunk function in patients with stroke or neurological disease. It includes turning to the affected/unaffected side, sitting up, and maintaining a seated position. Each item is scored on a 3-point scale (0, 12, 25 points), with a total score ranging from 0 to 100 [18]. Higher scores indicate better trunk function, and TCT has demonstrated good reliability and validity [4].

Statistical analyses

The BBS is an observational tool used to assess static and dynamic balance in patients with neurological or musculoskeletal disorders [19]. It consists of 14 tasks, each scored from 0 to 4, with a total score of 0 to 56. Higher scores indicate better balance ability. The BBS is a standardized, reliable measure with strong psychometric properties for patients with stroke [20,21]. Balance ability in patients with stroke is linked to critical events, such as falls [22]. Furthermore, trunk function, as measured by the TIS, is considered to play a key role in maintaining and controlling standing balance [23]. Since the BBS evaluates various aspects of standing balance, we considered it clinically relevant to explore the relationship between TIS and BBS scores. Therefore, this study used the BBS, a standard balance assessment tool widely used internationally, as an external anchor to estimate the MIC for TIS and TCT. Patients with a BBS increase of ≥5 points between admission and follow-up were classified as the "important change group", while others comprised the "non-important change group". This classification was based on previous findings indicating that the MIC for the BBS is 5 points in cases of assisted walking after stroke [24]. Comparability was demonstrated by comparing the scores of TIS and TCT between groups. Furthermore, a sensitivity analysis was conducted by comparing the baseline characteristics between the included and excluded samples. Missing data were handled using listwise deletion. Only cases with complete data for the relevant variables were included in the statistical analyses.

All statistical analyses were performed using R for Mac Version 4.4.1 (R Foundation for Statistical Computing, Vienna, Austria), and a value of p<0.05 indicated statistical significance.

Responsiveness

Responsiveness was assessed by comparing TIS and TCT scores between the two groups using an unpaired t-test. The means and standard deviations for each group were calculated, and mean values with 95% confidence intervals were obtained by resampling 2000 times using the bootstrap method. Skewness for TIS and TCT scores was evaluated to check distribution bias, where values >1 indicate a floor effect, and values <-1 indicate a ceiling effect [25]. The number of patients with perfect and zero scores was recorded, and floor or ceiling effects were considered present if ≥15% of patients achieved maximum or minimum scores [26]. Correlation analysis between changes in TIS, TCT, and BBS scores was conducted using Spearman's rank correlation coefficient (ρ).

MIC Estimate

The MIC was estimated using the receiver operating characteristic-based method (MIC ROC), an anchor-based approach. The MIC ROC analysis calculated the Youden index from the results of the plotted ROC curves and used the optimal cutoff scores for each change in TIS and TCT to separate important change and non-important change groups. The AUC was used to assess discrimination accuracy. Several recommendations exist for estimating MIC. First, we confirmed whether the selected anchor, i.e., the BBS, was suitable as an index to capture important changes in the results of the TIS and TCT. According to Cohen's rule, if the result of the correlation analysis of the change between outcomes and anchors across time points is ≥0.3, it is considered appropriate to estimate the MIC [26]. Next, we checked whether the AUC was appropriate for estimating the MIC. An AUC ≥0.7 is considered appropriate for estimating the MIC [27]. Therefore, the MIC was calculated when both of these recommendations were met. When it was determined that the conditions for MIC estimation were met, sensitivity, specificity, positive predictive value, and negative predictive value were calculated in addition to AUC. Each value was calculated with 95% confidence intervals by resampling 2000 times using the bootstrap method. Furthermore, the value of half a standard deviation has been reported to correspond to the MIC in a previous study [28], and in this study, it was additionally calculated as 0.5×the standard deviation of the change in TIS from baseline to follow-up.

## Results

**Figure 1:**
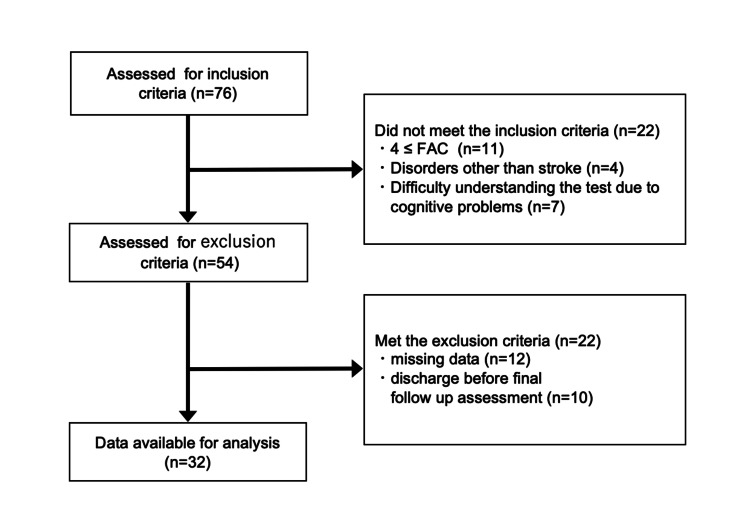
Flow diagram of the study sample of inpatients with subacute stroke requiring dependent ambulation FAC: Functional Ambulation Category

Figure 1 shows the flow diagram of the study sample, and Table 1 presents the clinical characteristics of the study sample. This study included a sample of 32 participants, many of whom were older and had suffered from cerebral infarction. The majority of patients had moderate to severe balance disorders. As a result of the sensitivity analysis comparing the baseline characteristics between the included and excluded samples, significant differences were observed in body weight (p=0.034) and the proportion of cerebral infarction (p=0.010), whereas no significant differences were found in other variables such as age, MMSE, or BBS.

**Table 1:** Clinical characteristics of the study sample SD: standard deviation; MMSE: Mini-Mental State Examination; FAC: Functional Ambulation Category; BBS: Berg Balance Scale; TIS: Trunk Impairment Scale; TCT: Trunk Control Test

Variables	Values
Age (years), (SD)	75.4 (10.8)
Height (m), (SD)	1.6 (0.1)
Weight (kg), (SD)	48.7 (7.6)
Gender (male), n (%)	15 (46.9)
Type of stroke (cerebral infarction), n (%)	21 (65.6)
Time since stroke (days), (SD)	25.1 (16.3)
Duration from baseline to follow-up (days), (SD)	33.2 (3.2)
Affected side (Rt), n (%)	15 (46.9)
MMSE (points), (SD)	21.0 (6.3)
FAC (0/1/2/3/4/5), n	10/8/6/9/0/0
BBS (points), (SD)	
Admission	23.1 (17.8)
Follow-up	29.0 (19.0)
Change score	5.9 (6.0)
TIS (points), (SD)	
Admission	9.4 (6.6)
Follow-up	11.5 (7.8)
Change score	2.1 (3.3)
TCT (points), (SD)	
Admission	66.3 (33.7)
Follow-up	76.9 (27.9)
Change score	10.5 (17.6)

Responsiveness

Regarding responsiveness, Table 2 shows the comparison of TIS and TCT scores and their distribution between the two groups. The difference between the important change group and the non-important change group was significant for the TIS change score (p=0.001), but not for the TCT change score (p=0.968). The results of the skewness analysis indicated that the distribution at both baseline and follow-up tended to be more skewed in TCT than in TIS. When examining perfect and zero scores, we found a floor effect for the TIS only at admission, with no ceiling effect. In contrast, the TCT exhibited a ceiling effect at both baseline and follow-up.

**Table 2:** Comparison of TIS and TCT between the two groups in inpatients with subacute stroke requiring dependent ambulation TIS: Trunk Impairment Scale; TCT: Trunk Control Test The values ​​are shown as mean±standard deviation (upper and lower limits of the 95% confidence interval for the mean). Statistics were performed using unpaired t-tests. The 95% confidence interval was calculated by resampling 2000 times using the bootstrap method. Floor effect is the percentage of participants with the lowest possible score, while ceiling effect is the percentage of participants with the highest possible score.

	Important change group (n=17)		Non-important change group (n=15)		P-value	Skewness	Floor effect (%)	Ceiling effect (%)
TIS (points)								
Admission	10.1±5.2	(7.5-12.5)	8.7±8.0	(4.8-12.7)	0.579	-0.025	15.6	0.0
Follow-up	13.8±6.8	(10.6-17.0)	8.9±8.2	(4.9-13.0)	0.071	-0.125	9.4	3.1
Change score	3.8±3.5	(2.2-5.4)	0.1±1.6	(-0.7-0.9)	0.001	-	-	-
TCT (points)								
Admission	81.2±26.0	(68.1-92.6)	49.5±34.3	(33.0-66.5)	0.006	-0.6	3.1	37.5
Follow-up	91.6±13.7	(84.6-97.4)	60.2±30.8	(44.4-75.5)	0.001	-1.1	3.1	43.8
Change score	10.4±16.3	(3.1-18.6)	10.7±19.5	(1.9-21.5)	0.968	-	-	-

**Figure 2:**
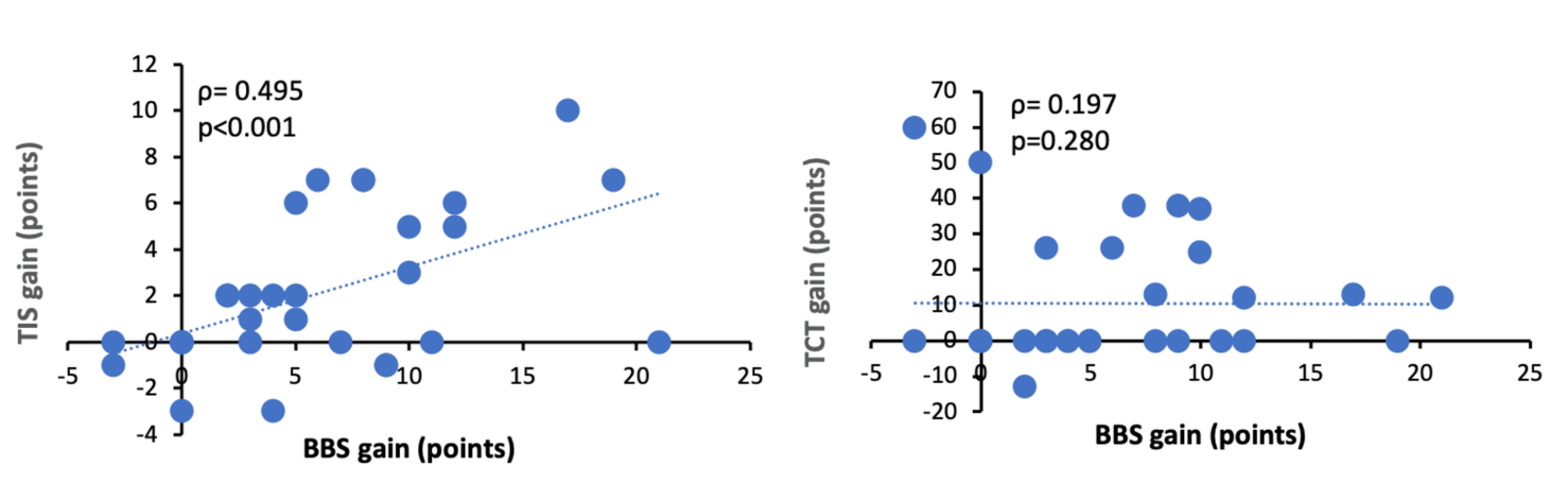
Results of the correlation analysis of change scores between TIS, TCT, and BBS in inpatients with subacute stroke requiring dependent ambulation BBS: Berg Balance Scale; TIS: Trunk Impairment Scale; TCT: Trunk Control Test Spearman's rank correlation (rs) was used for correlation analysis.

Figure 2 shows the results of the correlation analysis of change scores between the TIS, TCT, and BBS. A significant correlation was observed for the TIS (ρ=0.495; p<0.001), but no significant correlation was found for the TCT (ρ=0.197; p=0.280).

MIC

**Figure 3:**
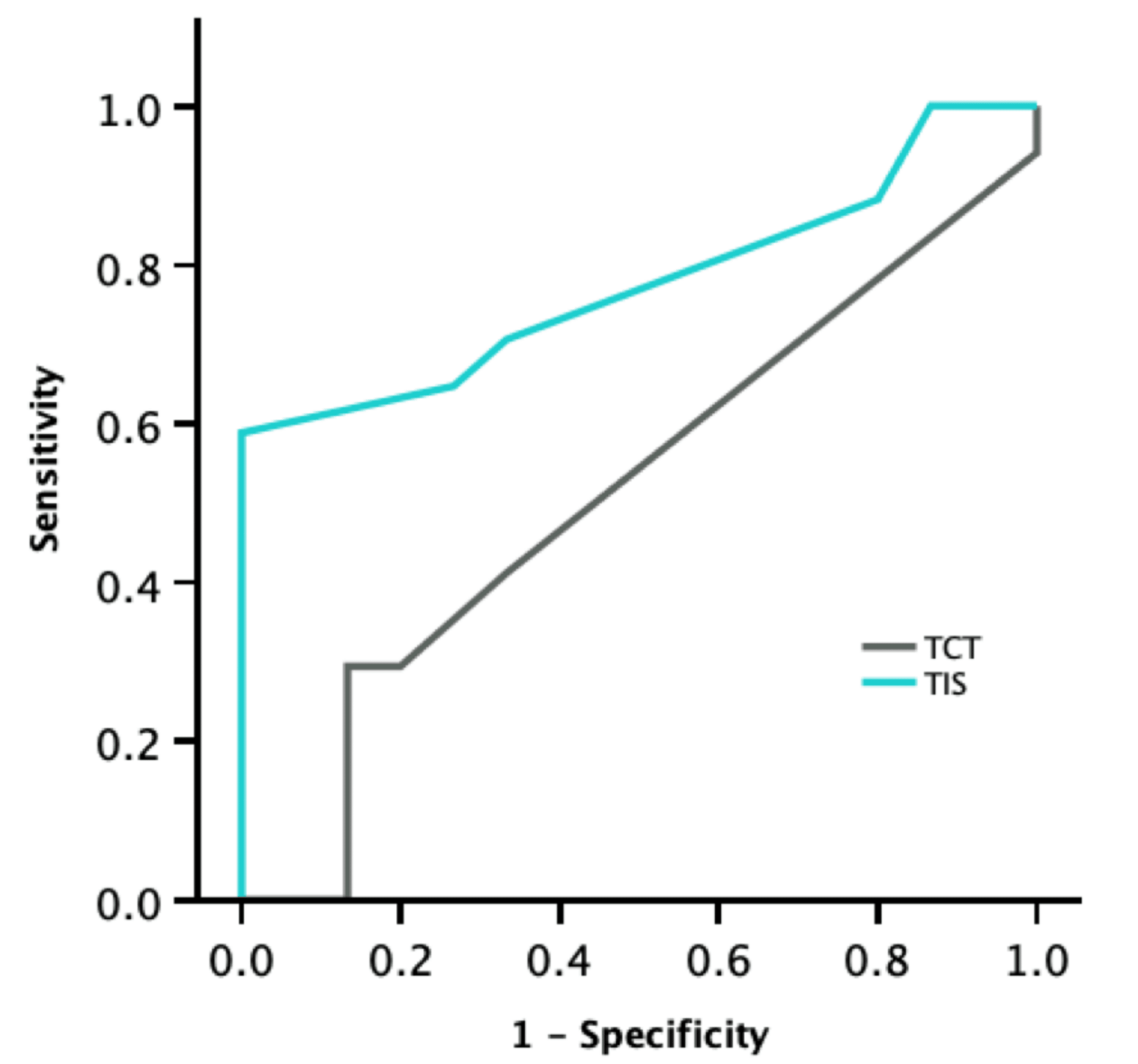
Results of calculating TIS and TCT using receiver operating characteristic curve in inpatients with subacute stroke requiring dependent ambulation TIS: Trunk Impairment Scale; TCT: Trunk Control Test

Figure 3 presents the results of the ROC curve analysis for the TIS and TCT. The AUC (95% CI) of the ROC curve for TIS was 0.776 (0.612-0.941), while the AUC (95% CI) of the ROC curve for TCT was 0.518 (0.313-0.722). Since only TIS met the criterion of AUC ≥0.7, MIC was estimated solely for TIS. Table 3 shows the results of the MIC analysis for the TIS. The MIC (95% CI) for TIS was 2.5 (0.5-4.0) points. Additionally, the MIC was calculated using the distribution-based method as 0.5 times the standard deviation, resulting in a value of 1.64 points.

**Table 3:** Results of minimal important change on TIS in inpatients with subacute stroke requiring dependent ambulation TIS: Trunk Impairment Scale; MIC: minimal important change; AUC: area under the curve; PPV: positive predictive value; NPV: negative predictive value The 95% confidence interval was calculated by resampling 2000 times using the bootstrap method.

	Values	95% CI	
		Lower	Upper
TIS			
MIC	2.50	0.50	4.00
AUC	0.776	0.612	0.941
Sensitivity	0.588	0.353	0.882
Specificity	1.000	0.800	1.000
PPV	1.000	0.813	1.000
NPV	0.682	0.577	0.846

## Discussion

This study is the first to investigate the responsiveness and MIC of the TIS, focusing specifically on inpatients with subacute stroke requiring assistance for ambulation. In this study, we also compared TIS with TCT, an existing method of measuring trunk function, to determine whether TIS is suitable for assessing intervention effectiveness. As a result, the MIC of the TIS calculated in this study confirmed the robustness of the MIC reported in a previous study [10]. Furthermore, TIS demonstrated superior responsiveness and interpretability compared to TCT, indicating that it may be suitable for assessing changes in trunk function in inpatients with subacute stroke requiring assistance for ambulation. Conversely, the traditionally used TCT may be less responsive and interpretable than the TIS, given that it showed a ceiling effect and failed to reach the AUC ≥0.7 threshold.

TIS may outperform TCT in assessing trunk function because it provides a detailed evaluation of static and dynamic balance as well as trunk coordination. In the present study, changes in TIS scores were significantly correlated with changes in BBS scores, suggesting that TIS is an important measure associated with improvements in trunk function and balance ability. A previous study investigating the MIC of TIS reported values of 3.5 points in patients with acute stroke and 2.5 points in patients with chronic stroke [10]. In those studies, the Global Rating of Change (GROC) was used as an anchor to evaluate patients' subjective changes from baseline to one month later. However, it should be noted that the inclusion criteria in previous studies were broad, such as "ability to sit", which may limit generalizability.

A unique feature of this study is that it specifically focused on patients who required either physical assistance or supervision for ambulation, used the BBS rather than the GROC as an anchor to assess balance ability, and employed multiple methods to estimate the MIC. The MIC of TIS in this study was calculated to be 2.5 points, which is generally consistent with a previous study [10], supporting its robustness. Calculating MIC in a targeted population provides a concrete criterion for evaluating patient improvement and is useful in clinical practice.

On the other hand, while the TCT is simpler and easier to administer, it appeared to exhibit a ceiling effect at both baseline and follow-up, which is consistent with previous reports [5]. This observation may indicate potential limitations in its ability to detect change, even among patients with relatively severe impairments who require assistance with ambulation following stroke. In addition, changes in TCT scores did not show a significant correlation with changes in BBS scores and fell short of the commonly referenced AUC ≥0.7 threshold. Taken together, these findings suggest that the TCT might be less sensitive in capturing balance-related changes in this patient population. By contrast, the present results may support the tentative usefulness of the TIS in assessing trunk function. The TIS showed signs of responsiveness to intervention and may serve as a helpful tool for monitoring functional changes over time. Furthermore, the MIC value derived from the TIS could potentially aid in the clinical interpretation of score changes and contribute to the formulation of individualized rehabilitation plans.

This study has several limitations. It was conducted at a single institution with a small sample size, which may limit generalizability. Although the sample size was sufficient for ROC curve analysis, it was smaller than the sample size recommended by COSMIN for MIC calculation. However, internal validation using bootstrapping was performed, which is considered to have enhanced the reliability of the results. Future studies should focus on external validation to confirm the generalizability of the MIC. The use of BBS as an external anchor may also limit interpretability, as BBS does not directly reflect subjective patient-reported outcomes such as the GROC. Nevertheless, balance ability is an important functional domain in stroke patients, influencing both fall risk and quality of life. For these reasons, the BBS is considered a valid and objective anchor for MIC calculation, capable of reflecting meaningful improvements that are less subject to bias. In addition, although this study targeted stroke patients broadly, it included individuals with subarachnoid hemorrhage, whose recovery trajectories may differ from those with cerebral infarction or intracerebral hemorrhage. This heterogeneity in stroke subtypes may have influenced the generalizability of the findings and should be taken into account when interpreting the results. Additionally, although the study population was limited to patients with FAC ≤3, there exists a notable functional gap between those at FAC level 3 and those at FAC levels 2 or below, particularly in terms of whether direct physical assistance is required for ambulation. This difference may influence responsiveness and MIC estimates, as well as clinical interpretation. However, the current study did not stratify participants by FAC level, which may limit the precision of the findings. Future studies should consider stratified analyses to account for this heterogeneity and to better reflect the clinical nuances among different levels of gait dependence. Although each assessment tool used in the study has been previously validated with regard to reliability, it should be noted that inter-rater reliability was not independently verified within the context of this study. Evaluations were conducted based on the assumption that the established reliability of these tools remains consistent in our clinical setting. Another point of consideration is the timing of the initial assessment, which was set within two weeks of admission. While this timing was chosen to minimize confounding due to fluctuations in consciousness level and timing of rehabilitation initiation, it may have introduced variability in participants' baseline status. Lastly, a comparison of the baseline characteristics between included and excluded participants revealed significant differences in body weight and the proportion of cerebral infarction. This discrepancy may reflect a selection bias, and caution should be exercised when generalizing the results to a broader stroke population.

## Conclusions

In this study, the MIC of the TIS in patients with subacute stroke who required assistance for ambulation was estimated to be 2.5 points. The TIS appeared to demonstrate greater responsiveness and a lower ceiling effect compared to the TCT. By re-examining the MIC of the TIS in a more specifically defined patient population, the present findings may offer clinically meaningful insights that could support more individualized patient evaluation and treatment goal-setting. Further research is warranted to more fully explore the potential utility of the TIS and to build a stronger evidence base for enhancing stroke rehabilitation outcomes.
